# Inhibiting the NLRP3 Inflammasome with MCC950 Alleviates Neurological Impairment in the Brain of EAE Mice

**DOI:** 10.1007/s12035-023-03618-y

**Published:** 2023-09-13

**Authors:** Baohua Hou, Jun Yin, Shuyan Liu, Jincheng Guo, Baobao Zhang, Zhenzhen Zhang, Lanping Yang, Xiying Tan, Yijiao Long, Sijie Feng, Jingchun Zhou, Yifan Wu, Xueyang Wang, Song Han, Zhenhui Wang, Xiaohua He

**Affiliations:** 1 College of Medicine, Henan Polytechnic University, Jiaozuo, 454000 China; 2 Department of Pathophysiology, School of Basic Medical Sciences, Wuhan University, Wuhan, 430000 China; 3 Central Laboratory, The First Affiliated Hospital of Henan Polytechnic University (Jiaozuo Second People’s Hospital), Jiaozuo, China; 4 Department of Endocrinology, The First Affiliated Hospital of Henan Polytechnic University (Jiaozuo Second People’s Hospital), Jiaozuo, 454000 China; 5 Department of Thoracic Surgery, The First Affiliated Hospital of Henan Polytechnic University (Jiaozuo Second People’s Hospital), Jiaozuo, 454000 China; 6 Beijing Bencaoyuan Pharmaceutical Co, Ltd, Beijing, 102629 China

**Keywords:** Multiple sclerosis, NLRP3, Microglia, Astrocyte, Experimental autoimmune encephalomyelitis

## Abstract

Multiple sclerosis (MS) is a chronic disease that is characterized by demyelination and neuronal damage. Experimental autoimmune encephalomyelitis (EAE) mice are used to model the disease progression of MS and mirror MS-like pathology. Previous researches have confirmed that inhibition of NLRP3 inflammasome significantly alleviated the severity of EAE mice and the demyelination of spinal cord, but its effect on neuronal damage and oligodendrocyte loss in the brain remains unclear. In this study, female C57BL/6 mice were immunized with MOG35–55 and PTX to establish experimental autoimmune encephalomyelitis (EAE) model. MCC950, a selective NLRP3 inflammasome inhibitor, was used to investigate the effect of NLRP3 inflammasome on the pathological changes and glial cell activation in the brain of EAE mice by immunohistochemistry. Our results demonstrated that MCC950 ameliorated the neuronal damage, demyelination, and oligodendrocyte loss in the brain of EAE mice. This protective effect of MCC950 may be attributed to its ability to suppress the activation of glial cells and prevents microglia polarization to M1 phenotype. Our work indicates that inhibition of NLRP3 inflammasome has the therapeutic effects of neuroprotection through immunomodulation and is a promising therapeutic strategy for MS.

## Introduction

Multiple sclerosis (MS) is considered an immune-mediated neurodegenerative disease of the central nervous system (CNS), with onset at 20–40 years of age [1]. Inflammation, demyelination, gliosis, oligodendrocyte death, and neuronal damage of the brain and spinal cord are the most pathological hallmarks of MS [2]. Although several drugs have been successfully developed to ameliorate symptoms and slow the progression of neurological disability, unfortunately, there are no effective therapies to halt the progression of MS [3, 4]. Therefore, there is still an urgent need to develop new agents for MS patients. Experimental autoimmune encephalomyelitis (EAE) mice are induced by the myelin oligodendrocyte glycoprotein (MOG) 35–55 peptide and Pertussis toxin (PTx), which is used to model the disease progression of MS and mirror MS-like pathology [5, 6]. Most researches focus on the pathological changes in the spinal cord of EAE mice, but sometimes the pathological changes in the spinal cord may not coincide with those in the brain [7]. In this research, we focused on the pathological changes in the brain of EAE mice, especially neuron damage and oligodendrocytes loss.

NLRP3 inflammasome is an intracellular multiple-protein complex in the innate immune cells, which is formed by NOD-like receptor containing pyrin-domain 3 (NLRP3), apoptosis-associated speck-like protein containing CARD (ASC), and proteolytic enzyme Caspase-1 [8, 9]. Activated NLRP3 inflammasome induces the maturation and secretion of IL-1β and IL-18 [8]. In neurodegenerative diseases such as Alzheimer’s disease [10, 11], Parkinson’s disease [12], amyotrophic lateral sclerosis [13], and MS [14, 15], dysregulation of this complex can trigger and sustain NLRP3 inflammasome activation and thereby drive CNS neuropathology. NLRP3 inflammasome might be a potential target for the treatment of inflammatory diseases [9, 11, 16]. In 2015, Coll and colleagues discovered that MCC950, a compound containing diphenyl sulfonylurea, is known to inhibit the activation of the NLRP3 inflammasome [17]. Previous researches have demonstrated that MCC950 significantly improved the severity of EAE mice and the demyelination of spinal cord [17], but its effect on neuronal damage and oligodendrocyte loss in the brain remains unclear.

In the present study, MCC950 was used to investigate the effect of NLRP3 inflammasome on the pathological changes in the brain of EAE mice, especially neuron damage and oligodendrocytes loss. In addition, we examined the effect of NLRP3 inflammasome on the activation of glial cells. These findings will help us find out the role of NLRP3 inflammasome in MS and provide new strategies for the treatment.

## Materials and Methods

### Animals

Six-week-old female C57BL/6 mice were obtained from Animal Experiment/ABSL-3 Laboratory and kept in an SPF facility at Wuhan University Center. All experimental procedures complied with the Committee on the Ethics of Animal Experiments of Wuhan University (China) (permit number: 2017083). The mice were grouped and kept under standard laboratory conditions (a 12-h light/dark cycle with an average room temperature of 25 °C and a relative humidity of 55–60%). The mice were provided food and water available ad libitum during the study. The animal room was cleaned regularly during the holding period. All methods were performed in accordance with the relevant ethical guidelines and regulations. This study complies with the ARRIVE guidelines [18].

### EAE Induction and Treatment

The EAE model was induced as previously described [19]. Put simply, female C57BL/6 mice were subcutaneously immunized with 200 μl of 100 μg myelin oligodendrocyte glycoprotein (MOG35–55, Wuhan Haode Peptide, China) in incomplete Freund’s adjuvant (Sigma Aldrich, USA) containing 200 μg mycobacterium tuberculosis (strain H37Ra; Difco, USA) on day 0. Pertussis toxin (PTX; List Biologicals, USA) (200 ng) was administered intraperitoneally (i.p.) on day 0 and 2. EAE scores were evaluated as followed: 0.5, partial tail limpness; 1, tail limpness; 1.5, reversible impaired righting reflex; 2, impaired righting reflex; 2.5, paralysis of one hindlimb; 3, paralysis of both hindlimbs; 3.5, paralysis of both hindlimbs and one forelimb; 4, hindlimb and forelimb paralysis; 5, death. Animals were scored daily by two independent investigators in a blind fashion.

The mice were randomly divided into three groups (*n* = 12 in each group): (1) the control group (Ctrl), mice received saline; (2) the EAE group (EAE), mice were received immunization; (3) the EAE + MCC950 group (EAE + MCC950), EAE mice were intraperitoneally injected with MCC950 (10 mg/kg, Medchem Express, China) at induction of the disease, days 0, 1, and 2 and every 2 days thereafter.

### Cell Culture

BV2 microglial cells were cultured in MEM media (HyClone, USA) containing 10% fetal bovine serum (FBS, Gibco, Brazil), 1% penicillin–streptomycin (Biosharp, China), and then incubated in a 37 ℃ incubator. BV2 microglial cells were treated with or without 100 ng/ml lipopolysaccharide (LPS, Sigma-Aldrich, St. Louis, MO, USA) in the absence or presence of 10 μM MCC950 for 24 h.

### Tissue Preparation

Twenty days after immunization, the EAE mice reached peak onset and showed obvious motor dysfunction [20]. Then, mice were deeply anaesthetized under 1.5% isoflurane (RWD, Shenzhen, Guangdong, China), and transcardially perfused with 50 ml of phosphate-buffered saline (PBS) followed by 50 ml of 4% paraformaldehyde (PFA). Brain and spinal cord tissues were dissected and fixed in PFA overnight at 4 °C.

### Hematoxylin and Eosin Staining

To evaluate the severity of inflammation, hematoxylin and eosin staining was performed as previous described [21]. Fixed spinal cords were paraffin-embedded and cut into 4-μm sections by paraffin slicer (Leica RM2016). The sections were stained with hematoxylin–eosin (H&E, Servicebio, China) and photographed with an Olympus AH-2 light microscope (× 200; Olympus, Japan). The severity of inflammation was scored according to previously published modified criteria on H&E stains [21]: 0, no infiltrate or focal meningeal infiltrates; 1, sparse infiltrates in the parenchyma; 2, severe perivascular infiltrate foci in the parenchyma with involvement of < 5% of the white matter; 3, invasions involving 5 to 25% of the white matter; and 4, diffuse infiltration involving > 25% of the white matter.

### Luxol Fast Blue Staining

To evaluate the severity of demyelination, Luxol fast blue staining was performed as previous described [20]. The 4-μm sections were stained with Luxol fast blue (LFB, Servicebio, China)) and photographed with an Olympus AH-2 light microscope (× 200; Olympus, Japan). Demyelination score was evaluated using the following scale described by previously published [20]: 0, no demyelination; 1, a few, scattered naked axons; 2, small groups of naked axons; 3, large groups of naked axons; and 4, confluent foci of demyelination.

### Immunohistochemistry

Fixed brains were paraffin-embedded and cut into 4-μm sections. After antigen retrieval, the sections were blocked and stained with anti-Oligo2 (1:500, Proteintech), anti-MBP (1:200, Abcam), anti-NG2 (1:100, Millipore), anti-Iba1 (1:500, Proteintech), anti-GFAP (1:500, Proteintech), anti-MAP2 (1:500, Proteintech), anti-PSD95 (1:500, Cell Signaling Technology), or anti-Synapsin I (1:500, Cell Signaling Technology). Then, the sections were incubated with a horseradish peroxidase (HRP)-conjugated anti-rabbit antibody (1:500, Abbkine, China) or (HRP)-conjugated anti-mouse antibody (1:500, Abbkine, China). The sections were developed using DAB peroxidase substrate (Beyotime Biotechnology, China). The sections were photographed with an Olympus AH-2 light microscope (× 200; Olympus). Images were imported into ImageJ for quantification. Briefly, the well-stained area on the images was first selected; then, the mean integrated optical density value of the area was calculated by ImageJ. According to the mean integrated optical density of the control group, the relative density of the EAE group and the MCC950 treatment group was calculated.

### Immunofluorescence

The BV2 microglial cells were fixed with 4% PFA and blocked with 3% bovine serum albumin (BSA) for 1 h with gentle rocking. Then, the cells were incubated for 12 h at 4 °C with the primary antibody iNOS (1:500, Proteintech). After washing with PBS, Cy3-labeled-IgG (Wuhan goodbio technology, China) were used as secondary antibodies. Cell nuclei were visualized with DAPI (Sigma-Aldrich, St. Louis, MO, USA). Images were visualized with an Olympus AH-2 light microscope (× 200; Olympus). Images were imported into ImageJ, and the relative density of the three group was calculated by the same method as immunohistochemistry.

### Golgi Staining

To evaluate the severity of neuronal damage, Golgi staining was performed according to the instructions (Servicebio, China). Briefly, fixed brains were washed with PBS several times and incubated in Golgi-Cox solution for 14 days. Then, the brains were sliced at a thickness of 100 μm and collected for silver staining. The images were photographed with an Olympus AH-2 light microscope (Olympus). The length of axon was calculated by ImageJ. The skeletonized image of the neuron and dendritic spine were created by Fiji. The number of spines was also calculated by Fiji.

### Data Analysis

All data are expressed as the mean ± standard error of the mean (SEM). Statistical analysis was performed using the GraphPad Prism software (version 7, USA). Statistical differences among the three groups were determined using one-way ANOVA with the Newman–Keuls test. Statistical differences between two groups were analyzed using Student’s *t*-test. For all analyses, statistical significance is denoted as *p < 0.05, **p < 0.01, or ***p < 0.001.

## Result

### Inhibition of NLRP3 Inflammasome Ameliorates Pathological Changes in the Spinal Cord of EAE Mice

**Fig. 1:**
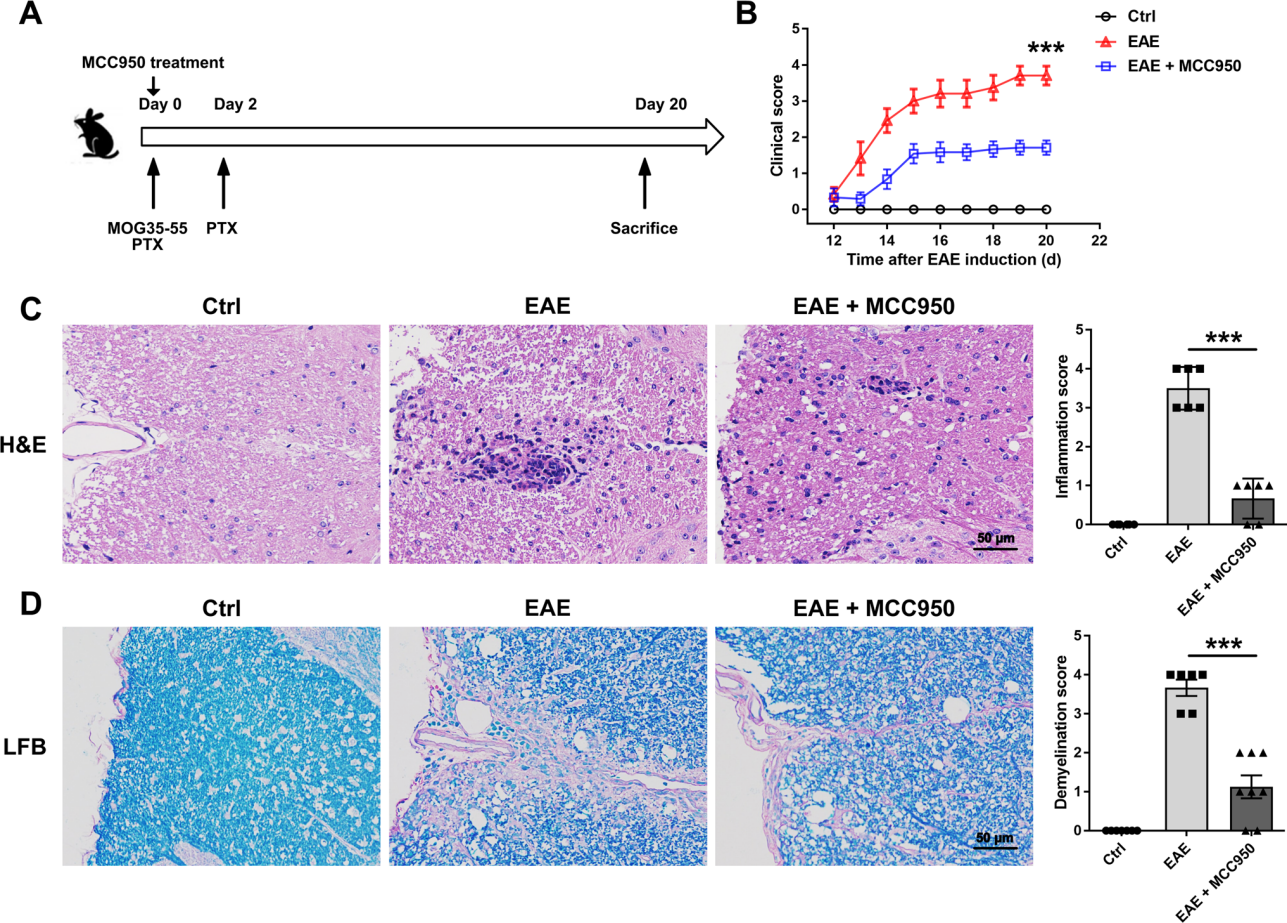
MCC950 ameliorates inflammation and demyelination in the spinal cord of EAE mice. **A** A schematic illustration of EAE progression. **B** Clinical scores of C57BL/6 mice treated with PBS or MCC950. **C** Representative H&E staining and inflammation score in the spinal cord of different groups. **D** Representative LFB staining and demyelination score in the spinal cord of different groups. Data represent the mean ± SEM, *n* = 12, *p < 0.05, **p < 0.01, ***p < 0.001

At first, the clinical scores were observed longitudinally. As shown in Fig. 1B, treatment with MCC950 significantly reduced the severity of EAE. Then, we investigated the effect of MCC950 on EAE severity using hematoxylin–eosin (H&E) and Luxol fast blue (LFB) staining. Compared to control, extensive inflammatory cells were detected around the white matter of the spinal cord from the EAE mice (Fig. 1C). Moreover, a large amount of demyelination was observed in the spinal cord of EAE mice, especially in white matter (Fig. 1D). However, treatment with MCC950 showed a reduction in inflammation and demyelination (Fig. 1C, D). These results indicate that the EAE model was made, and MCC950 can indeed alleviate the severity of EAE mice.

### Inhibition of NLRP3 Inflammasome Ameliorates Neuronal Damage in the Brain of EAE Mice

**Fig. 2:**
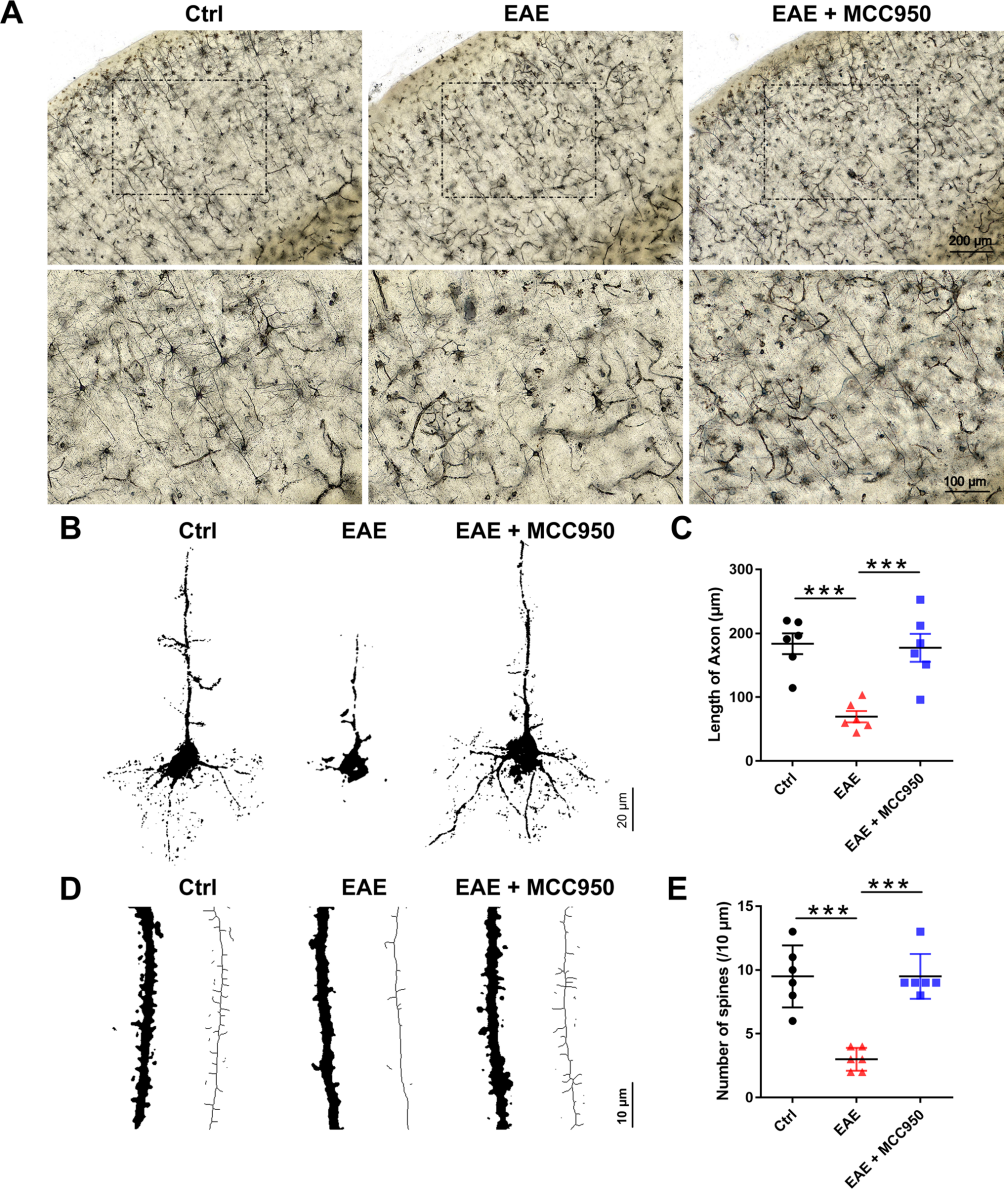
MCC950 ameliorates neuronal damage in the brain of EAE mice at the morphological level. **A** Representative Golgi staining of the cortex region in the different groups. **B** Representative graphical drawing showing neuron in the different groups. **C** The length of axon was quantified in different groups. **D** Graphical drawing showing dendrite in the different groups. **E** The spine number was quantified in different groups. Data represent the mean ± SEM, *n* = 3, *p < 0.05, **p < 0.01, ***p < 0.001

Furthermore, Golgi staining and immunohistochemical staining were used to assess the effect of NLRP3 inflammasomes on the neuronal damage of brain. Golgi staining was performed to visualize the entire structure of a neuron with a clear background and make it possible to investigate the neuronal morphology under a microscope [22]. As shown in Fig. 2A, compared with control mice, distribution of neurons in cortex of EAE mice were sparse and disorderly, while MCC950 pretreatment ameliorated the neuronal damage. Moreover, we found that the length of axon (Fig. 2C) and the spine density (Fig. 2D, E) were significantly reduced in the EAE mice compared with the control mice, whereas pretreatment with MCC950 alleviated this decrease (Fig. 2B–E).

**Fig. 3:**
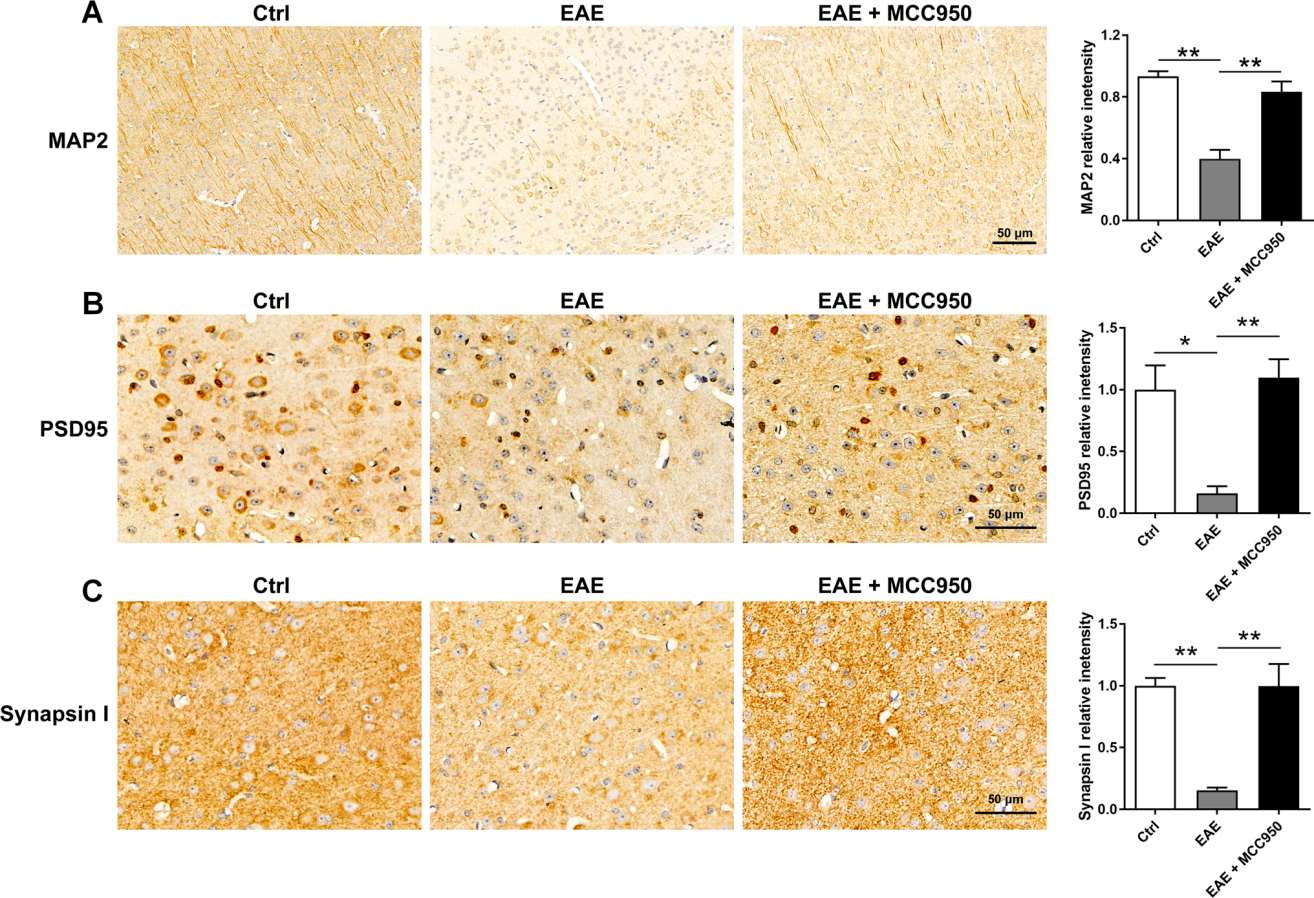
MCC950 ameliorates neuronal damage in the brain of EAE mice at the molecular level. **A** Representative immunohistochemistry of MAP2 expression and relative intensity in the cortex of different groups. **B** Representative immunohistochemistry of PSD95 expression and relative intensity in the cortex of different groups. **C** Representative immunohistochemistry of Synapsin I expression and relative intensity in the cortex of different groups. Data represent the mean ± SEM, 3 random images per section, and *n* = 3, *p < 0.05, **p < 0.01, ***p < 0.001

We also detected the expression of the neuronal marker MAP2, postsynaptic marker PSD95, and the presynaptic marker Synapsin I by immunohistochemistry, which were used for evaluation the effect of NLRP3 inflammasomes on the neuronal damage at the molecular levels. As shown in Fig. 3A, B, C, compared with control mice, the expression of MAP2, PSD95, and Synapsin I were remarkably reduced in the brain of EAE mice, while pretreatment with MCC950 restored the levels of these protein (Fig. 3A, B, C). The data above indicate that EAE mice exhibited obvious neuronal damage and synapse loss, while MCC950 treatment can ameliorate this damage.

### Inhibition of NLRP3 Inflammasome Ameliorates Demyelination and Oligodendrocyte Loss in the Brain of EAE Mice

**Fig. 4:**
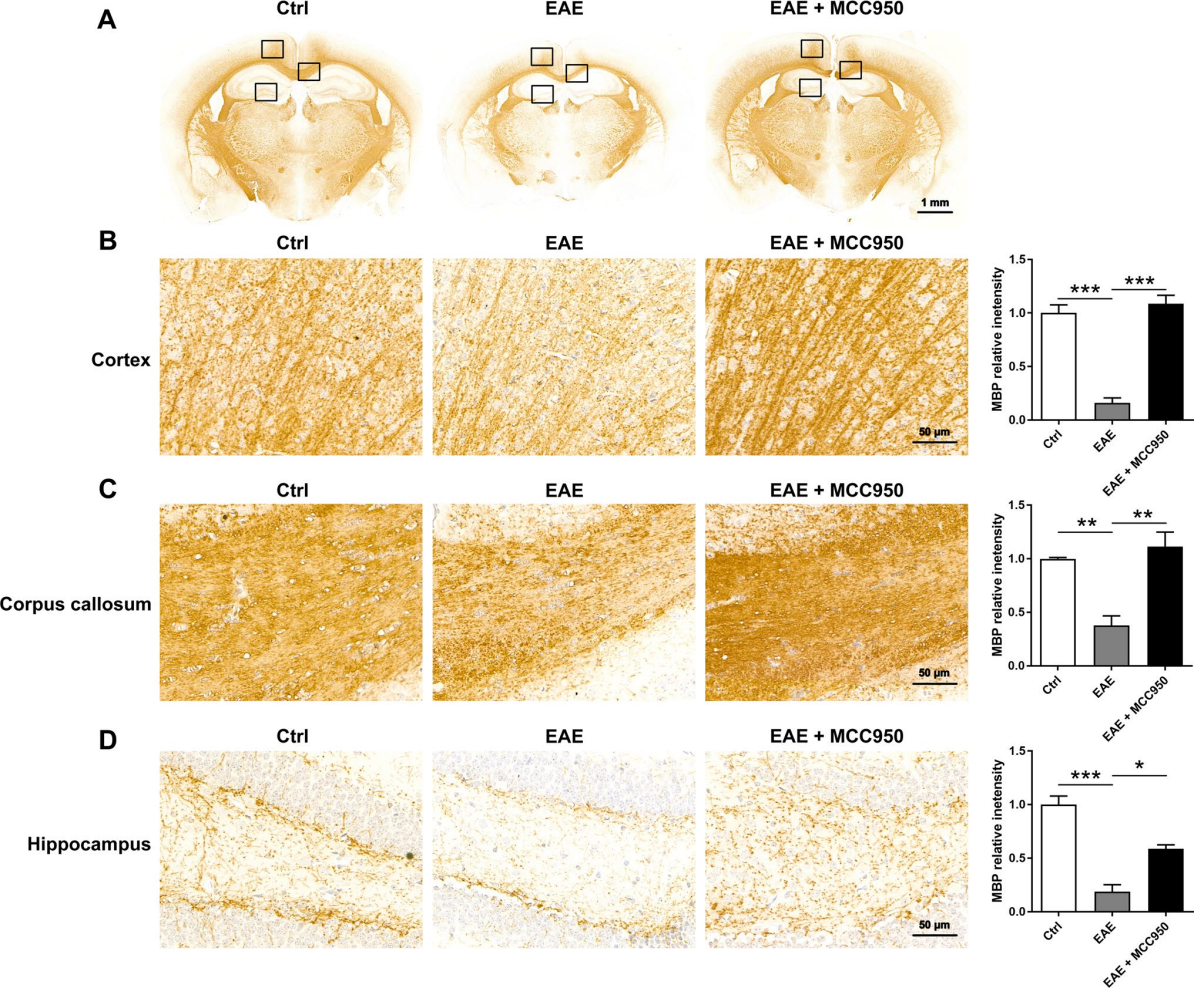
MCC950 reduces demyelination in the brain of EAE mice. **A** Representative immunohistochemistry of MBP expression of different groups. **B** Representative immunohistochemistry of MBP expression and relative intensity in the cortex of different groups. **C** Representative immunohistochemistry of MBP expression and relative intensity in the corpus callosum of different groups. **D** Representative immunohistochemistry of MBP expression and relative intensity in the hippocampus of different groups. Data represent the mean ± SEM, 3 random images per section, and *n* = 3, *p < 0.05, **p < 0.01, ***p < 0.001

Beside the neuron damage, we also performed the immunohistochemistry for myelin basic protein (MBP, mature oligodendrocyte markers) to evaluate demyelination in the brain. As shown in Fig. 4, compared with control mice, severe demyelination was observed in the brain of EAE mice, including the cortex (Fig. 4B), corpus callosum (Fig. 4C), and hippocampus (Fig. 4C), while MCC950 treatment exhibited remarkably reduced demyelination (Fig. 4).

**Fig. 5:**
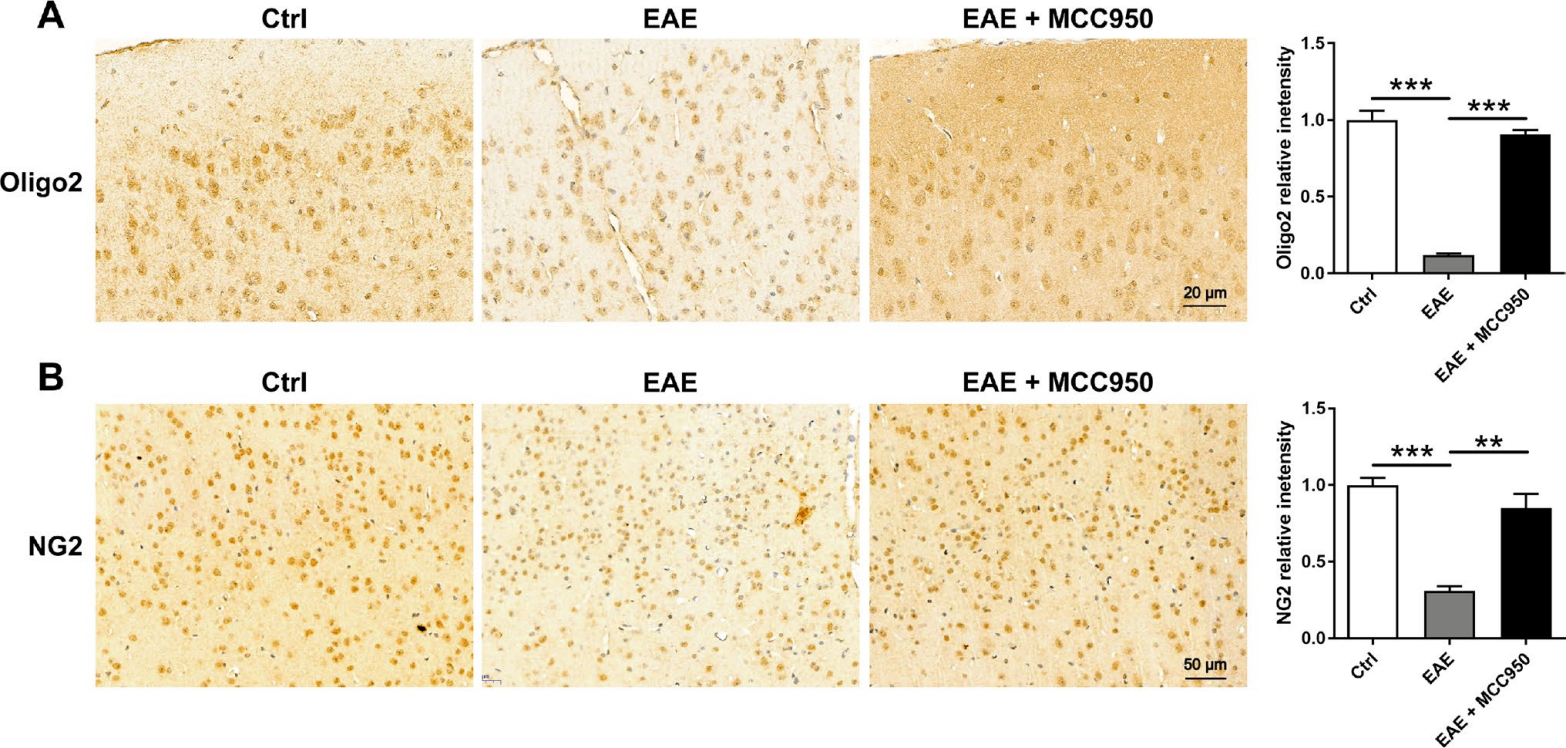
MCC950 reduces oligodendrocyte loss in the brain of EAE mice. **A** Representative immunohistochemistry of Oligo2 expression and relative intensity in the cortex of different groups. **B** Representative immunohistochemistry of NG2 expression and relative intensity in the cortex of different groups. Data represent the mean ± SEM, 3 random images per section, and *n* = 3, *p < 0.05, **p < 0.01, ***p < 0.001

We next investigated the effect of MCC950 on oligodendrocyte precursor cells (OPCs) using immunostaining for the OPC marker NG2 and Oligo2. As expected, compared with control mice, NG2^+^ cells (Fig. 5A) and Oligo2^+^ cells (Fig. 5B) densities were reduced in the brain of EAE mice, while MCC950 treatment exhibited a higher density of NG2^+^ cells and Oligo2^+^ cells (Fig. 5). The data above indicate that MCC950 treatment can ameliorate myelin loss in the brain of EAE mice.

### Inhibition of NLRP3 Inflammasome Reduces the Activation of Microglia and Prevents Microglia Polarization to M1 Phenotype

**Fig. 6:**
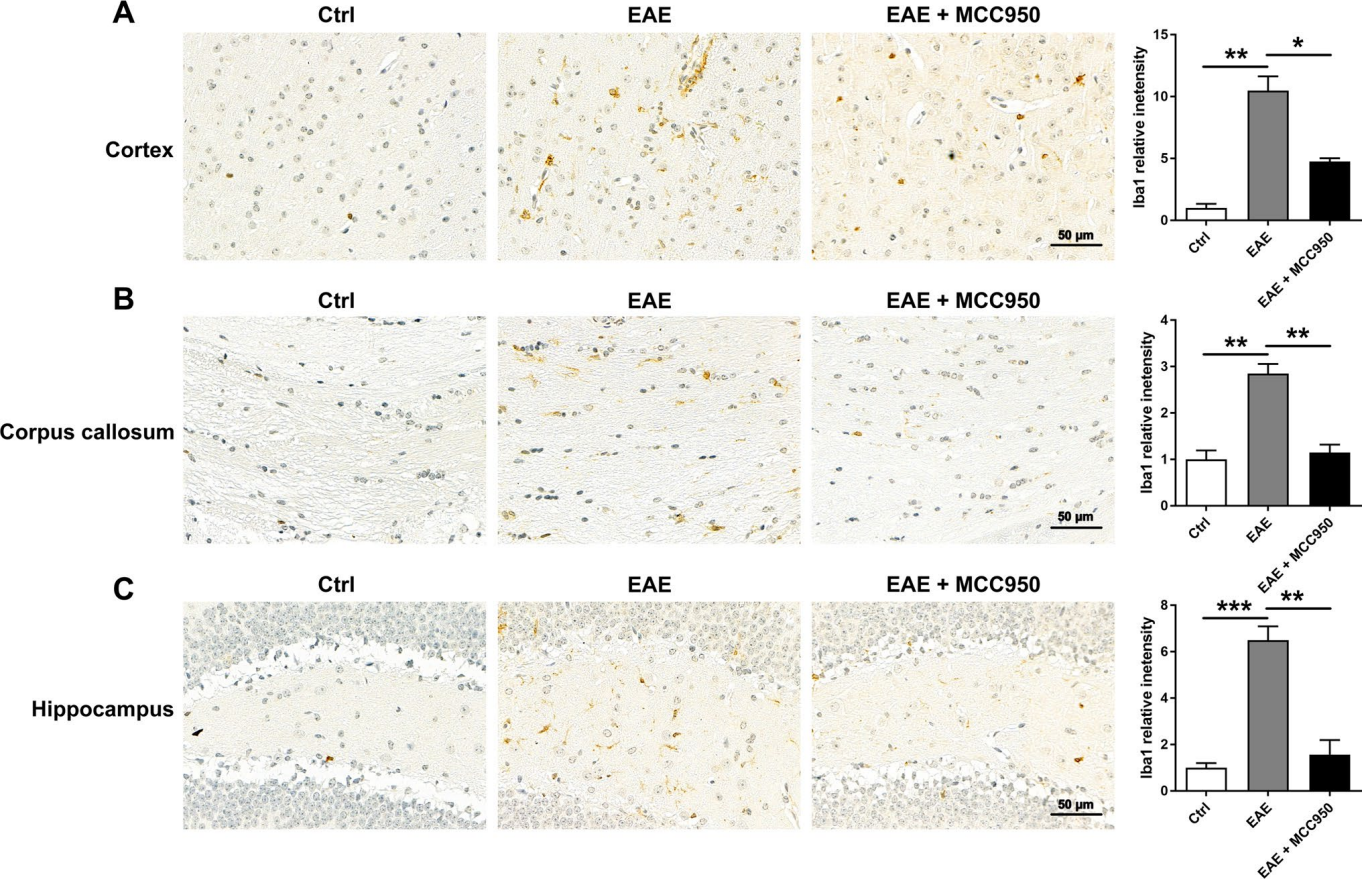
MCC950 reduces microglia activation in the brain of EAE mice. **A** Representative immunohistochemistry of Iba1 expression and relative intensity in the cortex of different groups. **B** Representative immunohistochemistry of Iba1 expression and relative intensity in the corpus callosum of different groups. **C** Representative immunohistochemistry of Iba1 expression and relative intensity in the hippocampus of different groups. Data represent the mean ± SEM, 3 random images per section, and *n* = 3, *p < 0.05, **p < 0.01, ***p < 0.001

**Fig. 7:**
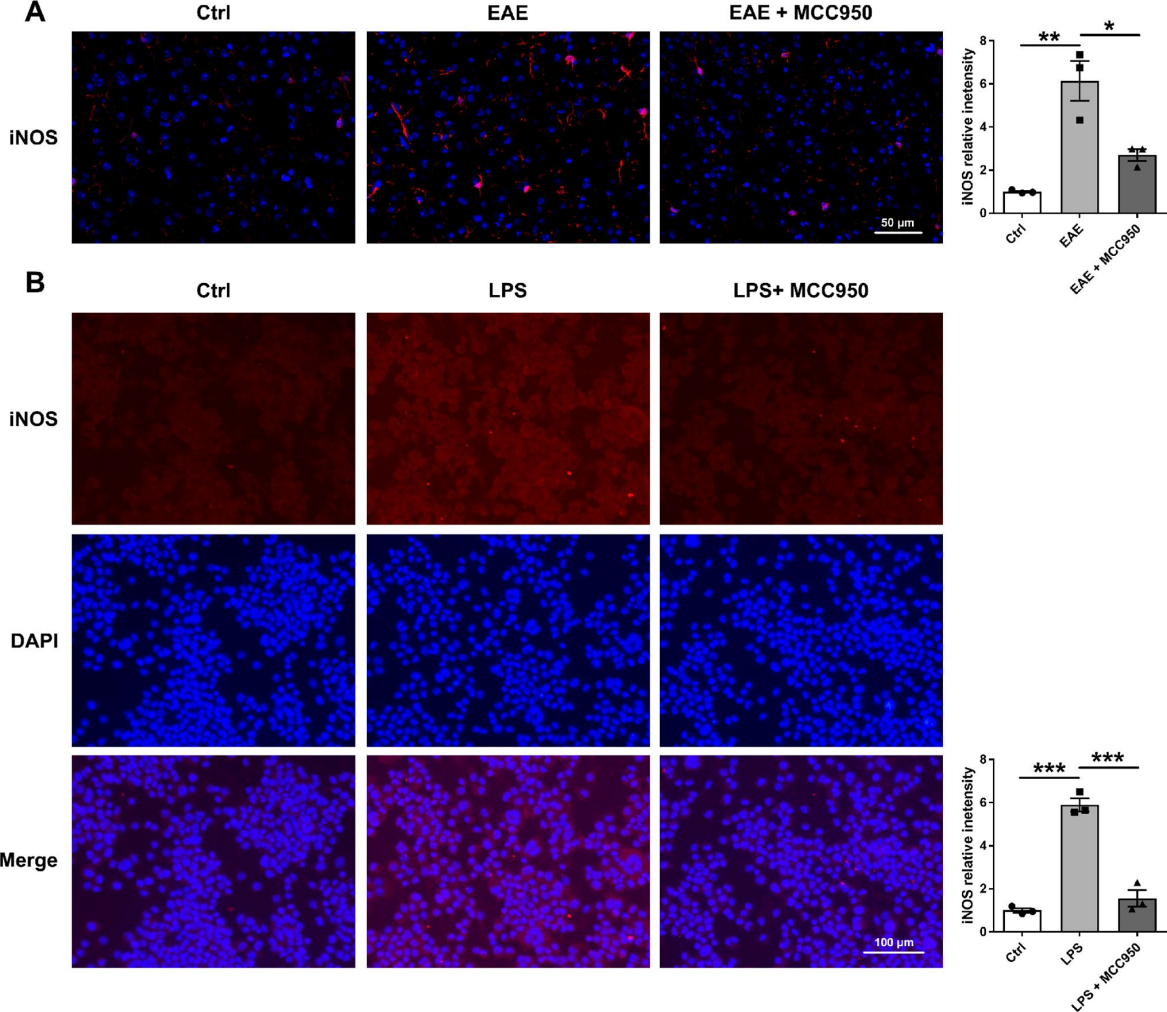
Inhibition of NLRP3 inflammasome prevents microglia polarization to M1 phenotype. **A** Representative immunohistochemistry of iNOS expression and relative intensity in the cortex of different groups. **B** Representative immunofluorescence of iNOS expression and relative intensity in BV2 cell culture. Data represent the mean ± SEM, 3 random images per section, and *n* = 3, *p < 0.05, **p < 0.01, ***p < 0.001

Microglia and astrocytes are important immune cells in CNS, and the activation of microglia is associated with neuronal damage and demyelination [23–26]. So, we performed immunohistochemistry for the microglia marker Iba1 to assess the activation change of microglia. As shown in Fig. 6, compared with control mice, we found that microglia were significantly activated in the brain of EAE mice, including the cortex (Fig. 6A), corpus callosum (Fig. 6B), and hippocampus (Fig. 6C), while MCC950 treatment remarkably reduced the activation of microglia (Fig. 6). Microglial activation is often categorized as either classical (the M1 phenotype) or alternative (the M2 phenotype). The M1 phenotype mainly induces neuroinflammation and neurotoxicity, whereas the M2 phenotype mainly releases prosurvival and neuroprotective factors [27]. Therefore, we also performed immunofluorescence for the M1 microglia marker inducible nitric oxide synthase (iNOS) to assess the phenotype change of microglia. As shown in Fig. 7A, compared with control group, the expression of iNOS was significant increase in the brain of EAE mice, while MCC950 treatment inhibited microglia polarization to M1 phenotype. We also examined the effect of MCC950 on microglia polarization using BV2 cells in vitro. After treatment with lipolysaccharide (LPS, Sigma), the BV2 cells acquired a pro-inflammatory M1 phenotype [28], and the expression of iNOS was increased (Fig. 7B). However, inhibition of NLRP3 significantly reduced the expression of iNOS (Fig. 7B).

### Inhibition of NLRP3 Inflammasome Reduces the Activation of Astrocytes

**Fig. 8:**
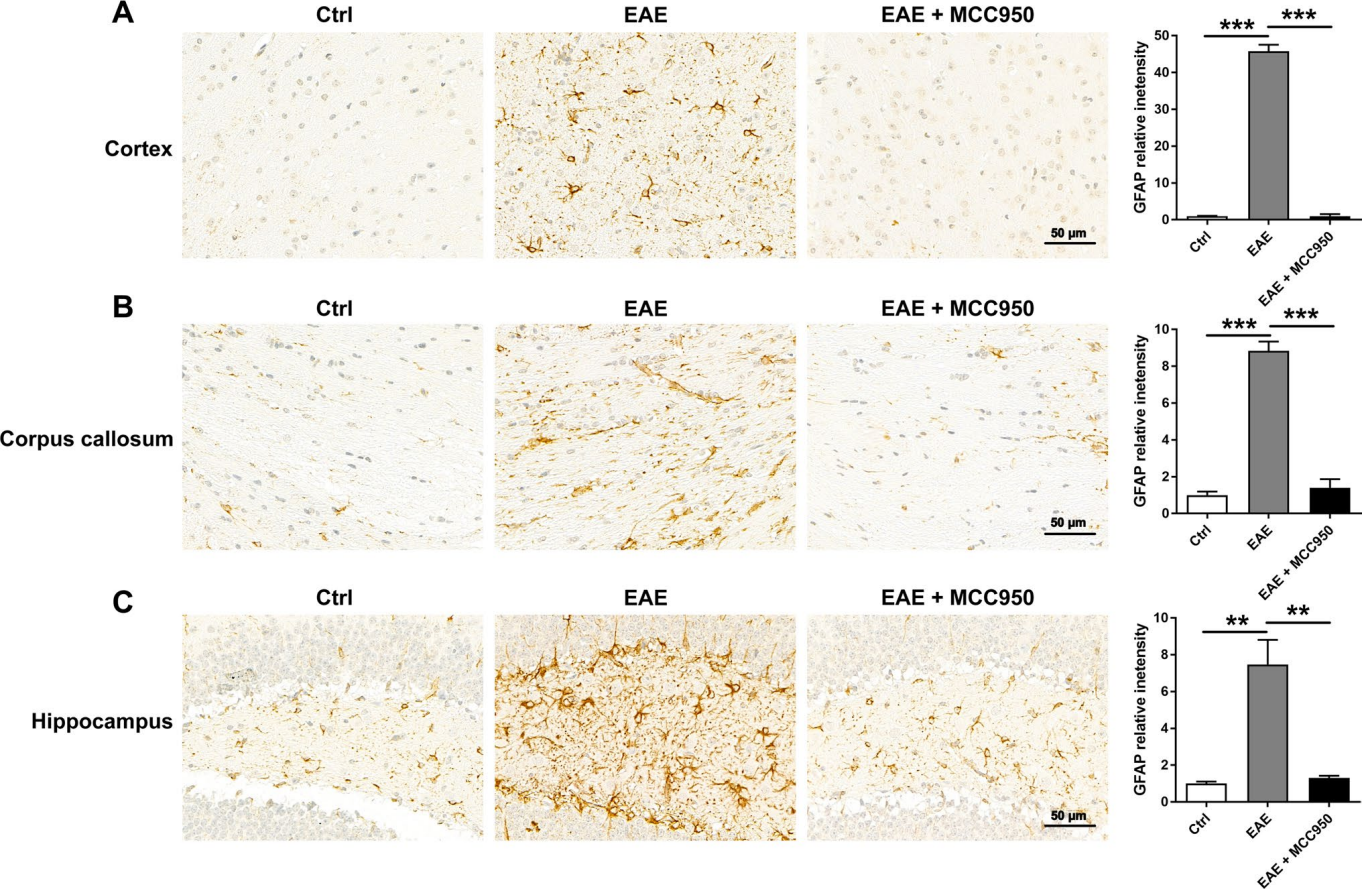
MCC950 reduces astrocyte activation in the brain of EAE mice. **A** Representative immunohistochemistry of GFAP expression and relative intensity in the cortex of different groups. **B** Representative immunohistochemistry of GFAP expression and relative intensity in the corpus callosum of different groups. **C** Representative immunohistochemistry of GFAP expression and relative intensity in the hippocampus of different groups. Data represent the mean ± SEM, 3 random images per section, and *n* = 3, *p < 0.05, **p < 0.01, ***p < 0.001

Microglia are the first cell type to respond following CNS disruption, and this is followed by reactive astrogliosis [29]. Astrogliosis is also involved in the development of EAE and MS [30]. Then, we examined the activation of astrocytes by immunohistochemistry for the astrocyte marker GFAP. As shown in Fig. 8, compared with control group, the astrocytes were significantly activated in the brain of EAE mice, including the cortex (Fig. 8A), corpus callosum (Fig. 8B) and hippocampus (Fig. 8C), MCC950 treatment remarkably reduced the activation of astrocyte (Fig. 8).

## Discussion

Multiple sclerosis is a chronic, inflammatory, and demyelinating disease of the central nervous system [31]. The pathological hallmark of MS is the accumulation of demyelinating lesions and neuronal damage in the brain and spinal cord [32, 33]. Most researches focus on the pathological changes in the spinal cord of EAE mice, but sometimes the pathological changes in the spinal cord may not coincide with those in the brain [7]. Brain pathology in MS patients is closely related to many complications, such as learning and memory impairment and depression [32, 34]. Therefore, exploring the brain pathological changes of MS model is important for exploring the therapeutic strategy of MS.

The activation of NLRP3 inflammasome has been demonstrated to contribute to pathology in a broad spectrum of neurological diseases, such as traumatic brain injury, spinal cord injury, Alzheimer’s disease, amyotrophic lateral sclerosis, and MS [35]. These researches indicated that NLRP3 inflammasome might be a potential target for the treatment of neurological diseases. Here, we focus on the effect of NLRP3 inflammasome on the pathological changes in the brain of EAE mice, especially neuron damage and oligodendrocytes loss. It has been reported that neuronal damage and synapse loss are found in the brain of EAE mice [36], which is consistent with our morphological and molecular results. Furthermore, we also found that inhibition of NLRP3 inflammasome decreased neuronal damage and synapse loss. In addition to neuronal damage, demyelination and oligodendrocytes loss are a recognized feature of EAE lesions [5]. In our study, we observed extensive loss of myelin and the reduction of OPCs in the brain of EAE mice. These results indicate that the brain of EAE mice show an impairment of the oligodendrocyte lineage. Meanwhile, significant decreases of neuronal damage and demyelination were detected with the MCC950 treatment, which indicated that NLRP3 inflammasome plays important role in pathogenesis of EAE mice.

In many brain disorders, microglia and astrocyte are activated and contribute to the progression of disease [13, 23, 37, 38]. In our study, we found that microglia are activated in the brain of EAE mice, while MCC950 can inhibit the activation of microglia, this was consistent with previous research [39]. Activated microglia are classed into two functional subtypes: M1 and M2 phenotypes [27]. M1 microglia release proinflammatory cytokines, such as interleukin-1 beta (IL-1β), IL-6, IL-12 and tumor necrosis factor (TNF), and high expression of iNOS. Consequently, a therapeutic approach targeting activated microglia or M1 microglia may be a promising strategy. In our research, we also found that NLRP3 inhibitor MCC950 could inhibit microglial M1 phenotype, which may form a suitable environment for neuron and oligodendrocyte.

Astrocytes are also involved in inflammatory response and phagocytosis. In our research, we also found that NLRP3 inhibitor MCC950 decreased astrocyte activation. Previous research found that the reactive astrocytes were termed A1 and A2, respectively [40]. A1 astrocytes are induced by classically activated neuroinflammatory microglia and shown to be destructive to neurons and oligodendrocytes [40]. So, the MCC950 may play an indirect inhibitory effect on astrocytes, which was due to inhibition of microglia. Interestingly, in previous research, we found that MCC950 could not reduce the activation of astrocytes [20], which was different from our present result. This was mainly due to the different timing of sampling in the two experiments, one at the peak phase (20 days after immunization) and the other at the late phase (40 days after immunization). We hypothesized that short-term inhibition of NLRP3 inflammasome might reduce the activation of A1 astrocytes, while long-term inhibition might promote the astrocytes transformation to A2 phenotype.

In summary, we show that MCC950, a selective blocker for the NLRP3 inflammasome, not only ameliorates pathological changes in the spinal cord of EAE mice but also prevents neuronal damage, demyelination, and oligodendrocyte loss in the brain. This protective effect of MCC950 may be related to its suppression of glial cell activation. Our work indicates that inhibition of NLRP3 inflammasome has the therapeutic effects of neuroprotection through immunomodulation and is a promising therapeutic strategy for MS.

## Data Availability

The datasets used and analyzed during the current study are available upon reasonable request from the corresponding author.
